# Anticancer Action of Silver Nanoparticles in SKBR3 Breast Cancer Cells through Promotion of Oxidative Stress and Apoptosis

**DOI:** 10.1155/2024/7145339

**Published:** 2024-02-19

**Authors:** Mohammad Vahabirad, Sajedeh Daei, Roghayeh Abbasalipourkabir, Nasrin Ziamajidi

**Affiliations:** ^1^Department of Clinical Biochemistry, School of Medicine, Hamadan University of Medical Sciences, Hamadan, Iran; ^2^Molecular Medicine Research Center, Hamadan University of Medical Sciences, Hamadan, Iran

## Abstract

Silver nanoparticles (AgNPs) are known as one of the highly utilized NPs owing to their unique characteristics in the field of cancer research. The goal of this research was to explore the oxidative stress, apoptosis, and angiogenesis in SKBR3 breast cancer cells after exposure to AgNPs. The survival rate of SKBR3 cancer cells and MCF-10A normal breast cells was assessed under the effects of different concentrations (0, 32, 64, 128, and 250 *μ*g/ml) by MTT method. The oxidative condition was assessed by measuring reactive oxygen species (ROS) production, total oxidant status (TOS), total antioxidant capacity (TAC), malondialdehyde (MDA), and antioxidant enzyme activity (CAT, GPx, and CAT) using colorimetric-based kits. Flow cytometry and Hoechst 33258 staining were performed to investigate the induction of apoptosis. Furthermore, the expression of Bcl-2-associated X protein (Bax), B-cell lymphoma 2 (Bcl-2), and caspase 3 and 7 activity was measured. The cell migration and vascular endothelial growth factor-A (VEGF-A) gene expression, protein kinase B (AKT), phosphatidylinositol 3-kinase (PI3K) were also studied. The MTT results indicated that AgNPs inhibit the SKBR3 cells' viability in a concentration-dependent way. Besides, AgNPs markedly induced oxidative stress via increasing TOS content, MDA production, reduction of TAC, and regulation of antioxidant enzyme level. Additionally, AgNPs promoted apoptosis as revealed by an enhancement in Bax/Bcl-2 expression ratio. Findings also indicated that AgNPs suppress the expression of genes (VEGF-A, AKT, and PI3K) involved in angiogenesis. Altogether, our data revealed that AgNPs initiate oxidative stress and apoptosis in SKBR3 breast cancer cells, dose dependently.

## 1. Introduction

Silver nanoparticles (AgNPs) have gained much attention in cancer research owing to their distinctive features and possible applications in cancer treatment. These nanoparticles, generally ranging from 1 to 100 nanometers in size, show excellent antibacterial, antifungal, and antiviral properties. Moreover, they have shown promising results in inhibiting the growth and proliferation of cancer cells [1]. Silver nanoparticles are introduced for use in cancer therapy because of their ability to selectively target cancer cells while sparing healthy normal cells. This selectivity is attributed to the unique surface properties of the nanoparticles, which enable them to interact with specific receptors on cancer cells [2]. Once attached to the cancer cells, AgNPs can induce various mechanisms by which prohibit their growth and eventually promote cell death [1].

One of the probable mechanisms by which AgNPs reveal their antitumoral action is through the generation of reactive oxygen species (ROS). These ROS molecules might cause oxidative stress within cancer cells, leading to DNA damage, protein denaturation, and ultimately cell death. Additionally, AgNPs have been shown to disrupt the mitochondrial function of cancer cells, further impairing their survival and proliferation [1, 3].

Breast cancer is one of the most frequent types of cancer which threatens women's life, worldwide [4]. Various strategies have been introduced to treat breast cancer, although mortality rates indicate the need for new treatment ways [5]. In recent publications, AgNPs have been introduced as a potential tool in the fight against breast cancer. These nanoparticles have shown promising results in inhibiting the growth and progression of breast cancer cells due to special characteristics [6, 7]. Despite the anticancer properties of these NPs, it has not yet been reported in clinical outcomes, although these NPs have the capability to use in creams and ointments for wound healing [8].

Apoptosis is a process of regulated cell death that is inhibited in tumoral cancer cells. B-cell lymphoma protein 2 (Bcl-2) and Bcl-2-associated X (Bax) proteins are known as anti- and proapoptotic regulators, respectively [9].

Angiogenesis is generally called the process of growth and creation of new vessels from existing vessels. Abnormal or increased angiogenesis is recognized as one of the characteristics of cancer [10]. Secretion of vascular permeability factor and vascular endothelial growth factor-A (VEGF-A) from tumoral cells is one of the main factors of angiogenesis. In addition, VEGF-A is a dimer glycoprotein and plays a central role in angiogenesis [11]. This glycoprotein together with a coreceptor called NRP1 (Neuropilin-1) forms a complex with VEGF receptors and causes angiogenesis in breast cancer [12]. Binding of VEGF to its receptor activates several downstream signaling pathways such as PI3K/Akt/mTOR pathways [13].

Previous publications have shown that AgNPs have an acceptable anticancer activity on breast cancer cells [8, 14–16]. Considering the high incidence of breast cancer, we decided to investigate the anticancer potential of AgNPs through oxidative stress and apoptosis pathway. To the best of our knowledge, this is the first study to explore oxidative stress and apoptosis in the presence of AgNPs under the same conditions in SKBR3 cells.

## 2. Materials and Methods

### 2.1. Chemicals

Polyvinylpyrrolidone-stabilized silver nanoparticle (AgNP) was purchased from NANOSANY Company (Mashhad, Iran). The characterizations of AgNPs were reported in our previous study [17]. Commercial kits including total antioxidant capacity (TAC), total oxidant status (TOS), malondialdehyde (MDA), superoxide dismutase (SOD), glutathione peroxidase (GPx), catalase (CAT), and caspases 3 and 7 were purchased from Kiazist Company, Iran. The cDNA synthesis kit was obtained from Pars Tous Company, Iran.

### 2.2. Cell Culture and Treatment

A flask containing SKBR3 cells was obtained from Iranian Genetic Resources Bank (Tehran, Iran) and transferred to the laboratory. The cells were incubated at 37°C with 5% CO_2_ pressure. After that, the cells were cultured in DMEM medium with 10% FBS and 1% antibiotic penicillin–streptomycin. The cells were passaged after 4-5 days, and the subsequent experiments were performed from the second to tenth passage.

AgNPs were diluted in distilled water in order to prepare the required solutions.

MCF-10A normal breast cells were also provided from Iranian Genetic Resources Bank to determine the probable cytotoxicity of the AgNPs.

### 2.3. Survival Rate of Cells by MTT Assay

In brief, the cells were placed in a 96-well plate at a concentration of 7 × 10^3^ cells per well. In the next day, the cells were exposed to the indicated dose of AgNPs (0, 32, 64, 128, and 250 *μ*g/ml). After completion of 24, 48, and 72 hours, MTT reagent was added to the wells and then placed in an incubator for 4 h. Subsequently, the content of wells was depleted, and DMSO substance was transferred to the wells. Finally, the absorbance was measured at wavelength of 570 nm with the aid of an ELISA reader (RT-2100C Microplate Reader, China).

### 2.4. Preparation of Lysate

In order to measure the activity of oxidative stress parameters, a number of 7 × 10^5^ SKBR3 cells were plated in each well of a 6-well plate, and after reaching the desired concentration, they were treated with the selected concentrations of AgNPs. After 24 h of incubation, the cells were washed with PBS. After trypsinizing the cells, they were centrifuged at 1500 rpm, and then, cold PBS buffer containing protease inhibitor was added to the cell pellet. After that, the cells were homogenized and freeze-thaw was performed three times to lyse the cells. Next, it was centrifuged for 15 minutes at 12000 rpm, and the supernatant was divided into new microtubes and kept at -70°C for further experiments.

### 2.5. Oxidative Stress Parameter Assay

Oxidative stress biomarkers including ROS, TAC, TOS, and MDA were determined with the aid of commercial kits following the manufacturer's recommendations (Kiazist, Iran). Also, the activity of antioxidant enzymes (GPx, CAT, and SOD) (Kiazist, Iran) was assessed by colorimetric reaction following the manufacturer's protocols which are explained in a previous publication [18]. It is worth mentioning that ROS production was measured via fluorimetric assay [19].

Protein content was determined by Bradford's assay [20] to normalize oxidative parameters.

### 2.6. Annexin/PI Apoptosis Assay

Apoptosis rate of SKBR3 cells was assessed by Annexin-V apoptosis assay kit based on the manufacturer's manual protocols and explanations provided from a previous report [21]. Briefly, the cells were placed in a 6-well plate and then treated with the NPs. After 24 h, the adherent cells (treated and control groups) were trypsinized and then collected and centrifuged for 5 min at 1500 rpm. The obtained cell sediment was dissolved in DMEM. After that, centrifugation was performed again, and the cell sediment was dissolved in 90 *μ*l of diluted Annexin-V binding buffer (1×). Then, we added 5 *μ*l of Annexin-V conjugate and 5 *μ*l of PI solution into the tube and maintained at dark place for 20 min. After this period, 400 *μ*l of Annexin-V binding buffer was transferred in to the tube and then centrifuged. Finally, the cell sediment was dissolved again in 500 *μ*l of the buffer, and all the samples were read on a flow cytometer (Life Technologies Attune NxT), and the obtained data were analyzed according to the unstained tube using FlowJo V10 CL software.

### 2.7. Hoechst 33258 Staining Assay

Morphological features of apoptosis were evaluated by DNA-binding fluorescent dye Hoechst 33258 staining. After fixation with methanol, the cells were stained with a working solution of Hoechst 33258 staining, placed at room temperature for 30 min, followed by observation on a fluorescence microscope (BEL, Italy).

### 2.8. Extraction of RNA from Cells and Quantitative Reverse Transcription-PCR (qRT-PCR)

In this research, we have evaluated the gene expression level of Bax, Bcl-2, VEGF-A, PI3K, and AKT where *β*-actin was regarded as a control. To do this, whole RNA was extracted from the cells by using TRIzol reagent, and then, cDNAs were synthesized using cDNA synthesis kit. Finally, the relative gene expression was examined using 2^−*ΔΔ*Ct^ method. The relative gene expression was reported as fold change with respect to the control. It should be said that the primer pairs were defined by Primer3 software (Table 1).

### 2.9. Measurement of Caspase 3 and 7 Activity

Caspase3 and 7 activity was assessed using a commercial kit from Kiazist Company. In this kit, the enzyme in each sample reacts with the common substrate of both caspase 3 and 7. Then, this enzyme breaks the substrate with a chromogen and produces a yellow color, followed by an evaluation of absorbance at the wavelength of 405 nm.

### 2.10. Scratch Wound Healing Assay

In brief, SKBR3 cells were grown at a density of 7 × 10^5^ cells per well in a 6-well plate. During the cultivation period, the amount of FBS was reduced to 2% in the culture medium in order to prevent the proliferation of cells. After 24 hours of incubation, a scratch was placed in the middle of the wells using a yellow tip. Then, the wells were washed with PBS to remove the dead and detached cells from the scratch. After that, the cells were exposed to the desired concentrations of AgNPs (0, 8, 16, and 32 *μ*g/ml) and were placed in an incubator for 24 hours. The cell migration was evaluated in the scratch space with the help of an inverted microscope (Nikon Eclipse TS 100) at zero and 24 h. Ultimately, ImageJ software was used to specify the migration area.

### 2.11. Statistical Analysis

GraphPad Prism 9 software was used for statistical analysis as well as drawing graphs. To perform the statistical analysis, the normal distribution of the data was first evaluated. Then, one-way ANOVA test was used to check the difference between the groups. The results were reported as mean ± standard error of the mean (SEM), in which the mean is the average of at least three replicates. Values less than 0.05 were considered statistically significant.

## 3. Results

### 3.1. Effects of Silver Nanoparticles on the Survival Rate of SKBR3 and MCF-10A Cells

**Figure 1:**
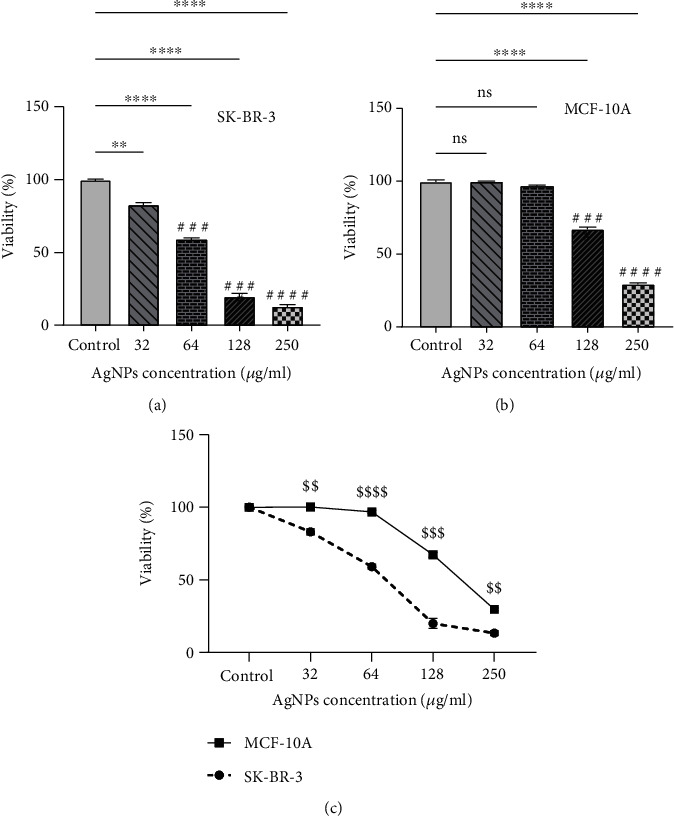
Anticancer action of silver nanoparticles (AgNPs) via inhibition of cell viability in SKBR3 cells after 24 h, adopted from MTT assay. The viability of exposed cells is reduced dose dependently. Results are expressed as mean ± SEM. ^∗,#^*P* < 0.05; ^∗∗,##^*P* < 0.01; ^∗∗∗,###^*P* < 0.001; ^∗∗∗∗,####^*P* < 0.0001. ^$$^*P* < 0.01, ^$$$^*P* < 0.001, and ^$$$$^*P* < 0.0001. ^∗^Compared to the control group. ^#^Compared to the 32 *μ*g/ml group. ^$^Compared to normal MCF-10A cells.

As it is shown in Figure 1(a), AgNPs inhibited the viability of SKBR3 breast cancer cells dose dependently (*P* < 0.05). Also, AgNPs reduced the viability of MCF-10A normal cells though its effects are meaningfully lower than in cancer cells (*P* < 0.05) (Figures 1(b) and 1(c)). As it is shown in Table 2, IC_50_ value was calculated as 63.01 ± 5.12, 61.77 ± 2.78, and 64.14 ± 3.29 *μ*g/ml after completion of 24, 48, and 72 h, respectively.

### 3.2. Oxidative Stress Findings

**Figure 2:**
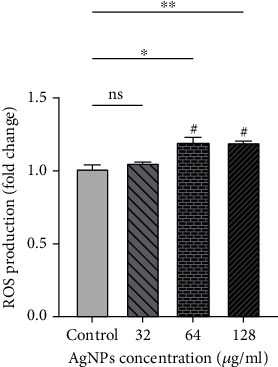
ROS production induced by AgNPs in SKBR3 cell. Results are reported as mean ± SEM (*n* = 3). ^∗,#^*P* < 0.05; ^∗∗^*P* < 0.01. ^∗^Compared to the control group. ^#^Compared to the 32 *μ*g/ml group.

The results show that ROS production was noticeably increased after exposure to concentrations of 64 and 128 *μ*g/ml AgNPs (*P* < 0.05) (Figure 2).

**Figure 3:**
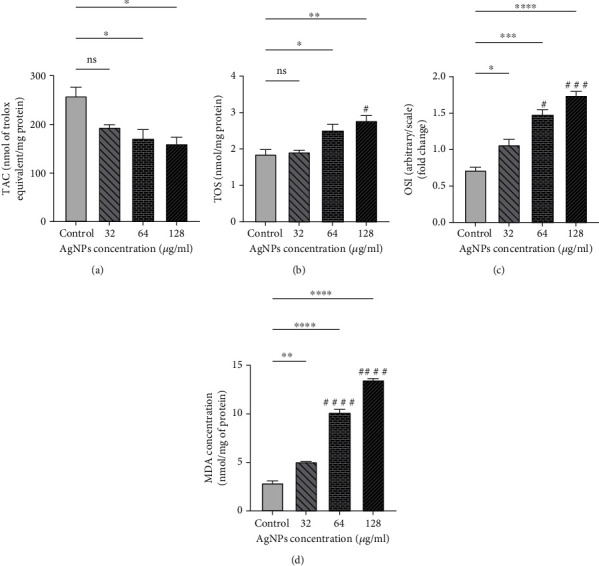
Oxidative stress biomarkers after being exposed to silver nanoparticles (AgNPs): (a) total antioxidant capacity (TAC), (b) total oxidant status (TOS), (c) oxidative stress index (OSI), and (d) malondialdehyde (MDA). AgNPs increased oxidative stress dose dependently, which is depicted in the figure. The results are reported as mean ± SEM in three replicates (*n* = 3). ^∗,#^*P* < 0.05; ^∗∗^*P* < 0.01; ^∗∗∗,###^*P* < 0.001; ^∗∗∗∗,####^*P* < 0.0001. ns: nonsignificant changes. ^∗^Compared to the control group. ^#^Compared to the 32 *μ*g/ml group.

Oxidative stress indicators including TOS, OSI, and lipid peroxidation (named as MDA) were considerably augmented in AgNP-treated cells at different levels of statistical significance (Figure 3). Additionally, TAC content was markedly reduced after exposure to the NPs (*P* < 0.05).

**Figure 4:**
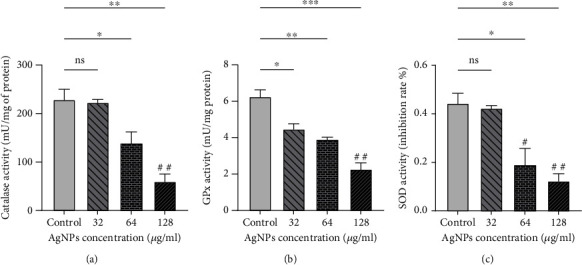
Antioxidant enzyme activity is decreased in SKBR3 cells after being exposed to silver nanoparticles (AgNPs): (a) catalase (CAT), (b) glutathione peroxidase (GPx), and (c) superoxide dismutase (SOD). ^∗,#^*P* < 0.05; ^∗∗,##^*P* < 0.01; ^∗∗∗^*P* < 0.001. ns: nonsignificant changes. ^∗^Significant changes compared to the control group. ^#^Significant differences compared with 32 *μ*g/ml group∗.

As it is represented in Figure 4, the activities of antioxidant enzymes including CAT, GPx, and SOD were also diminished in cells after treatment with AgNPs (*P* < 0.05). It is worth mentioning that these changes were dose dependent.

### 3.3. Apoptosis Observation by Hoechst 33258 Staining

**Figure 5:**
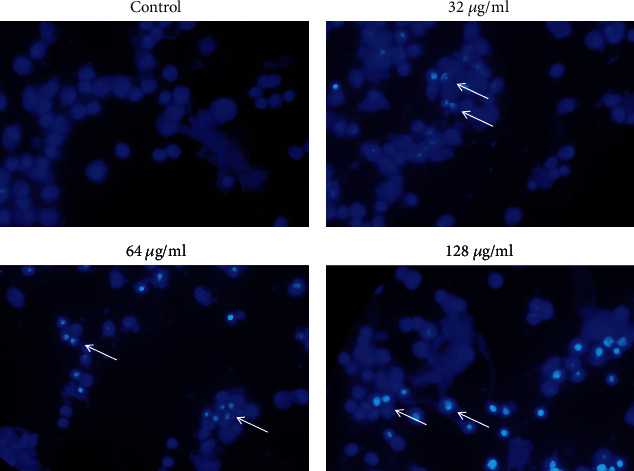
Apoptosis detection in SKBR3 cells by Hoechst 33258 dye. White arrows show cells with apoptotic morphology. Objective magnification is ×40.

Figure 5 shows the morphological changes in the nucleus of treated cells in which the sign of apoptosis was detected after Hoechst staining. It should be noted that the observations obtained from Hoechst staining in the indicated concentrations are consistent with the flow cytometry results.

### 3.4. Apoptosis Induction by AgNPs Using Annexin/PI Flow Cytometry

**Figure 6:**
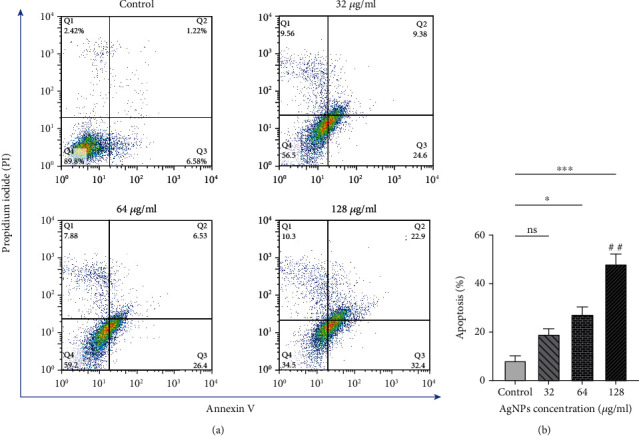
(a) Analysis of flow cytometry related to SKBR3 cells treated with silver nanoparticles for 24 h, obtained from Annexin-V/PI staining. Q1 quadrant shows necrotic cells stained by PI only, Q2 demonstrates late apoptotic cells, Q3 quadrant is early apoptotic, and Q4 quadrant contains viable cells. (b) Apoptosis rate of SKBR3 cells treated with silver nanoparticles which indicate increased apoptosis in 128 *μ*g/ml. The results are shown as mean ± SEM in three replicates (*n* = 3). ^∗^*P* < 0.05; ^∗∗,##^*P* < 0.01; ^∗∗∗^*P* < 0.001. ns: nonsignificant changes. ^∗^Compared to the control group. ^#^Compared to the 32 *μ*g/ml group.

As it is depicted from Figure 6, AgNPs promote apoptosis in SKBR3 cells as compared to the control untreated cells. The level of apoptosis was remarkably increased in the concentrations of 64 and 128 *μ*g/ml (*P* < 0.05), while the concentration of 32 *μ*g/ml could not significantly induce apoptosis in SKBR3 cells compared to the control group.

### 3.5. Apoptosis Detection by Caspase 3 and 7 Activity

**Figure 7:**
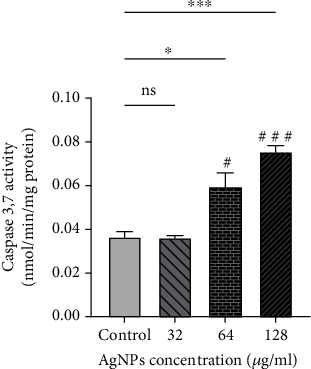
Caspase 3 and 7 activity increased after treating with silver nanoparticles (AgNPs) in 64 and 128 *μ*g/ml groups. ^∗,#^*P* < 0.05; ^∗∗∗,###^*P* < 0.001. ns: nonsignificant changes. ^∗^Compared to the control group. ^#^Compared to the 32 *μ*g/ml.

Based on the results shown in Figure 7, the activity of caspases 3 and 7 was not significantly changed in the 32 *μ*g/ml-treated group with respect to the control. However, Caspase 3 and 7 activity was significantly raised in SKBR3 cells after treatment with IC_50_ and higher doses of AgNPs (*P* < 0.05).

### 3.6. Gene Expression

**Figure 8:**
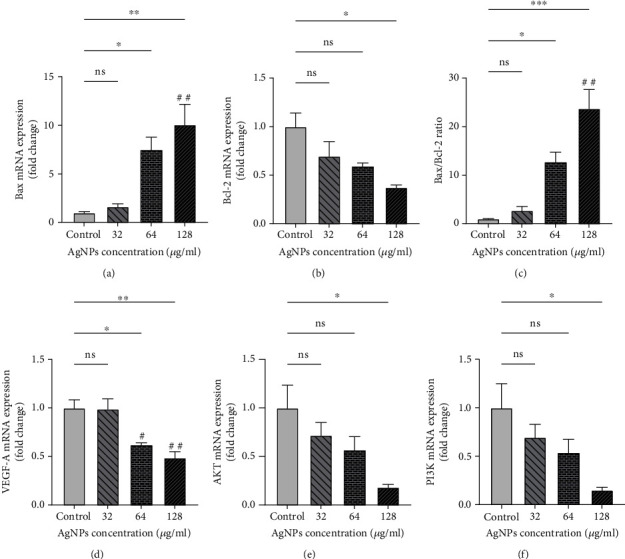
Gene expression level of (a) Bax, (b) Bcl-2, (c) Bax/Bcl-2, (d) VEGF-A, (e) AKT, and (f) PI3K in SKBR3 cancer cells after treating with silver nanoparticles (AgNPs). ^∗^*P* < 0.05; ^∗∗,##^*P* < 0.01; ^∗∗∗^*P* < 0.001. ns: nonsignificant changes. ^∗^Compared to the control group. ^#^Compared to the 32 *μ*g/ml group.

The results from qRT-PCR are shown in Figures 8(a)–8(f). Gene expression of Bax was meaningfully raised in the 64 and 128 *μ*g/ml groups compared to the control group. There were no significant differences in Bcl-2 gene expression at concentrations of 32 and 64 g/ml of AgNPs though the concentration of 128 *μ*g/ml significantly suppressed Bcl-2 gene expression compared to the control. The ratio of Bax/Bcl-2, considered as a marker of apoptosis, was significantly increased at the concentrations of 64 and 128 *μ*g/ml (*P* < 0.05). An enhancement of Bax/Bcl-2 ratio was also observed at the concentration of 32 *μ*g/ml compared to the control group, albeit it was not statistically significant (*P* > 0.05). Our results also showed that AgNPs decrease the expression of VEGF-A, PI3K, and AKT genes, which are known as regulators of angiogenesis pathway. As compared to the control cells, the VEGF-A gene expression was noticeably suppressed at the concentrations of 64 and 128 *μ*g/ml. The expression of AKT and PI3K gene was markedly decreased in the 128 *μ*g/ml dose of AgNPs with respect to the control. There was also an observed nonsignificant decrease in the concentrations of 32 and 64 *μ*g/ml relative to the control group.

### 3.7. Cell Migration in SKBR3 Cells

**Figure 9:**
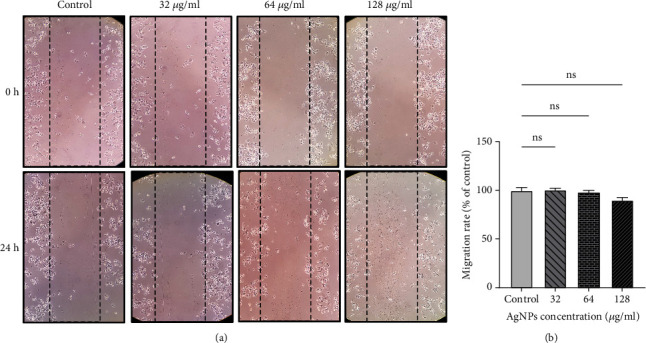
(a) Cell migration is shown in the image by wound healing assay and (b) migration rate of SKBR3 cells after exposure to silver nanoparticles (AgNPs). ns: nonsignificant changes.

Figure 9 shows the migration rate of SKBR3 cells after being exposed to various doses of AgNPs. The treated concentrations of AgNPs could not significantly reduce SKBR3 cell migration.

## 4. Discussion

Despite extensive efforts to find new therapeutic strategies, breast cancer remains one of the major causes of death in women, worldwide. Many preclinical evidences have shown that nanotechnology is very helpful in the therapeutic and diagnostic aspect of breast cancer [22]. To the best of our knowledge, the action of AgNP-induced SKBR3 cell toxicity is not well understood.

In this study, we have investigated the effects of AgNPs on the survival rate of SKBR3 breast cancer cell line and MCF-10A normal breast cells. We also investigated the effect of these NPs on ROS production, apoptosis, and angiogenesis in SKBR3 cells. Our results showed that the toxicity of NPs in SKBR3 breast cancer cells is much higher than normal breast MCF-10A cells after 24 hours of treatment. It has been demonstrated that different cancer cell lines exhibit higher sensitivity to AgNPs compared to normal cells, including MCF-10A [23]. In addition, our data emphasize that AgNPs induce ROS production in a dose-dependent way. Besides, to evaluate the oxidative damage caused by increased ROS level, the content of TAC, TOS, and MDA was investigated. In line with the results of the current study, Motafeghi et al. [24] observed that AgNPs promote ROS production, raise the level of MDA, and reduce glutathione (GSH) in MCF-7 breast cancer cells. Moreover, Al-kawmani et al. [25] observed an increased amount of ROS in AgNP-treated breast cancer cells. In our previous study, we also concluded that AgNPs enhance the level of TOS and MDA and reduce the activity of antioxidant enzymes in 5637 bladder cancer cells [17]. Further, Li et al. [26] found that AgNPs diminish SOD and GSH levels and increase MDA content which represent oxidative stress. In addition to oxidative stress, they also viewed a rise in Bax and a decrease in Bcl2 levels as apoptotic regulator.

According to the results of present report, AgNPs have the ability to attenuate the activity of antioxidant enzymes and change the oxidant/antioxidant status, which is consistent with the former investigations [17, 26, 27]. Similarly, in A2780 ovarian cancer cells, AgNPs decreased the activity of CAT and SOD enzymes and the level of glutathione. Additionally, a considerable increase in MDA levels indicates the onset of oxidative stress by AgNPs [28]. In line with our data, Panax ginseng Meyer-mediated AgNPs showed considerable anticancer effects on A549, MCF-7, and HepG2 cancer cells as revealed by heightened level of oxidative stress [29]. In contrast, some previous reports emphasized the antioxidant properties of AgNPs in which an enhancement of antioxidant enzymes such as SOD and CAT is reported [30]. In essence, the characteristic of nanoparticles might affect their final biological activity within cells [31]. Moreover, some plant extracts have anticancer activity though they are known as antioxidant agents [32, 33]. Though promising, a balance has not yet been found between the killing properties of pro-oxidant agents in cancer cells and their oxidative damage on normal tissues [34].

Flow cytometry and Hoechst staining showed that treatment with AgNPs induces apoptosis in SKBR3 cells in a dose-dependent manner. In agreement with the present study, Rajivgandhi et al. [7] have observed nuclear damage in MCF-7 cells treated by AgNPs after staining with Hoechst stains.

In this study, we have investigated the internal apoptosis or mitochondrial-dependent pathway of apoptosis. In this process, proapoptotic agents are released from the mitochondria under various signals, leading to the activation of caspases and ultimately cell death. The Bcl-2 protein family plays a crucial role in regulating this pathway, with members such as Bax and Bak promoting apoptosis, while others like Bcl-2 and Bcl-xL inhibit it [35]. Here, we found increased level of Bax as well as lower Bcl-2 gene expression and enhanced caspase 3 and 7 activity, which all confirm apoptosis occurrence in SKBR3 cells. In connection with this, Suseela et al. [36] expressed that AgNPs initiate apoptosis in A549 lung cancer cells using Annexin-V/FITC staining. It was also shown that AgNPs trigger apoptosis by increasing the amount of ROS and then changing the mitochondrial membrane potential of cells. In accordance with these results, the apoptosis-inducing ability of AgNPs in cancer cells was confirmed by recent reports [3, 37].

Our results revealed that AgNPs decrease the expression of VEGF-A, PI3K, and AKT genes, dose dependently. These genes play a substantial role in angiogenesis and metastasis of tumoral cells.

The PI3K/AKT has significant roles in the downstream signaling pathways of VEGF/VEGFR-2. This pathway is basically activated in many cancers, including breast cancer, and adjusts the migration of endothelial cells through VEGFR-2/PI3K/Akt/eNOS cascades [38–40].

In this context, the study conducted by Gurunathan et al. [41] showed that AgNPs inhibit the PI3K/AKT signaling pathway in eye endothelial cells and decrease the expression of VEGF to inhibit cell growth and migration. Likewise, previous research reported the downregulation of AKT in SKBR3 cells after exposure to AgNPs [14], albeit the nature of the AgNPs was different with the current study. Taken together, AgNPs hold great potential in breast cancer therapy through induction of oxidative stress and apoptosis.

Since regulation of redox homeostasis is of great importance in nanomedicine, future research is needed to perform vast experimental validation of redox medicine under AgNP therapy conditions.

## 5. Conclusion

In the current study, we assessed the anticancer effects of AgNPs on SKBR3 breast cancer cells. Our results showed that AgNPs have the potential to induce oxidative stress and cell death as revealed by increased level of oxidative markers, reduced antioxidant capacity, and upregulated Bax/Bcl-2 gene expression. Simultaneously, AgNPs suppressed the expression of VEGF-A, PI3K, and AKT genes, dose dependently. Despite the promising results of the current study, more research is still needed to better discover the process of action as well as possible side effects of AgNPs on normal cells. Additionally, optimizing their synthesis methods and determining the appropriate dosage for effective treatment are important considerations for their clinical translation.

## Figures and Tables

**Table 1:** Primer sequences used in qRT-PCR.

Gene	Forward	Reverse
*β*-Actin	5′-AAGATCAAGATCATTGCT-3′	5′-TAACGCAACTAAGTCATA-3′
Bax	5′-GGTTGTCGCCCTTTTCTACTT-3′	5′-GGAGGAAGTCCAATGTCCAG-3′
Bcl-2	5′-ATGTGTGTGGAGAGCGTCA-3′	5′-CAGCCAGGAGAAATCAAACA-3′
VEGF-A	5′-CTTGCCTTGCTGCTCTACCT-3′	5′-GTGATGATTCTGCCCTCCTC-3′
PI3K	5′-GGAAAGGTGGGAGGGGAGGT-3′	5′-TCTGAGGGTGAGGAAGGAGGT-3′
AKT	5′-GTGGGTATTGTGAAGGAGGGTTGG-3′	5′-AGCGGATGATGAAGGTGTTGGG-3′

**Table 2:** Inhibitory concentration (IC_50_) (*μ*g/ml) value of AgNPs in SKBR3 cell line.

IC_50_ (hours)	1^st^ IC_50_	2^nd^ IC_50_	3^rd^ IC_50_	Mean ± SEM
24 h	67.17	69.04	52.82	63.01 ± 5.12
48 h	63.53	56.32	68.57	61.77 ± 2.78
72 h	61.74	60.03	70.65	64.14 ± 3.29

## Data Availability

The data that support the findings of this study are available from the corresponding author on request.
